# Preventive effect of combined Er, Cr: YSGG and fluoride gel on acid resistance of the permanent tooth enamel: An *in vitro* study

**DOI:** 10.4317/jced.60023

**Published:** 2023-03-01

**Authors:** Dhuha-Najm Abdulhussein, Aseel-Haidar M.J Al Haidar

**Affiliations:** 1 Department of Pedodontics and Preventive Dentistry, College of Dentistry, University of Baghdad, Iraq

## Abstract

**Background:**

Increasing the resistance of enamel to acids may prevent dental erosion and diminish microhardness alterations in the enamel. This study aimed to evaluate the preventive effect of erbium, chromium: yttrium, scandium, gallium, and garnet laser combined with 1.23% acidulated phosphate fluoride gel on enamel resistance to demineralization.

**Material and Methods:**

Thirty-four human maxillary first premolars were randomly assigned to three groups. Group I (control group), group II were treated with fluoride gel for 4-minutes and group III received ten seconds of laser treatment; then fluoride has applied. Each sample was immersed in a soft drink for 2 minutes, then washed and kept in deionized water. Four consecutive cycles were carried out at 6 hours intervals. The effects were studied utilizing the Vickers microhardness test and scanning electron microscopy. Data analyses were performed by Levene test and General Linear Model Repeated Measure Factorial ANOVA with Bonferonni post hoc test, the accepted level of significance was 0.05.

**Results:**

Microhardness was increased statistically in groups II and III after treatment, with the highest value for group III. After demineralization, the control group had the lowest microhardness score, followed by group II and group III, which showed the least reduction in microhardness with a statistical significance (*p*<0.05). There was a correlation between the morphological alterations in enamel surfaces and the increased enamel resistance.

**Conclusions:**

Both fluoride and the combined laser fluoride treatment had an advantage in protecting the enamel and increasing enamel resistance to acids, with a more significant benefit for the combined laser fluoride group.

** Key words:**Prevention, Er, Cr: YSGG, enamel demineralization, fluoride, microhardness.

## Introduction

Eroded tooth structure is the result of enamel demineralization, which is a serious clinical problem (1,2) caused by exposure to intrinsic acids (such as in patients with eating disorders) or extrinsic acids(acidic food or beverages) in the absence of bacteria (3-5). While these defects are difficult to be noticed in their initial stages due to the presence of minute alterations to the tooth surfaces (6), in advanced cases, observation of concavities, alteration of the tooth anatomy as well as its colour can be detected (7).

The use of acidulated phosphate fluoride (APF) gel can prevent erosion by reducing the penetration of acids into the enamel (8,9); since it can aid in the formation of a superficial CaF2 layer (10). Yet, the efficiency of fluoride treatment in controlling dental erosion is restricted, which emphasizes the need for innovative technologies like laser therapy to prevent and control dental erosion.

Several theories tried to explain the mechanisms by which laser irradiation can enhance enamel resistance (11). Due to the fact that Er, Cr: YSGG laser (wavelength: 2780 nm) is well absorbed by water and the hydroxyl radical in hydroxyapatite, modern dental therapeutic strategies facilitate its use. Thus, it improves enamel acid resistance by causing enamel morphological and chemical changes without raising the heat (12-14).

A combination of fluoride and laser could stop erosion and minimize demineralization by many mechanisms, providing a long-lasting synergistic protective effect. Laser irradiation creates micro-spaces that can capture free fluoride ions that transform the hydroxyapatite crystals and yield fluorapatite, thus enhancing the enamel’s ability to resist demineralization (15).

The employment of Er, Cr: YSGG laser alone or in conjunction with fluoride components (comprising calcium and phosphate) and their efficacy according to the order of application in decreasing the solubility of enamel are still the topic of controversy in the scientific literature. Therefore, the aim of this study was to assess the synergistic effects of Er, Cr: YSGG and 1.23% APF gel in protecting enamel and inhibiting demineralization.

## Material and Methods

-Sample size

It was calculated prior to the research using the G power software (3.1.9.7) with a 0.31 effect size, 80% power of the study, two-sided test at 5% alpha error of probability. With three groups, a minimum of 8 samples for each group was rounded to 10 to account for dropouts. Another four teeth were added for SEM analysis (representative samples not included in statistical analysis comprised of one sound sample and one sample from each study group after demineralization with a soft drink (untreated control group, fluoride gel only, and combined laser + fluoride gel).

-Sample preparation 

Thirty-four human maxillary first premolars (extracted for orthodontic treatment) were chosen according to the selection criteria: sound crowns, absence of restorations, cracks, fractures, or other developmental defects in the tooth. The teeth were kept in a 0.1% solution of thymol (M Dent, Bangkok, Thailand) at room temperature until they were used to keep teeth moist and avoid dehydration, reduce enamel brittleness, and fungal and bacterial development on teeth (4).

Polyvinyl plastic cylinder tubes (Zhejiang Liutong plastics co., Ltd., China) were cut into equal rings with a 20 mm diameter and 8 mm depth with parallel and flat top and bottom sides to make samples suiTable for the micro-hardness test. After that, each ring was filled with cold cure acrylic resin (Akrodent, Ankara, Turkey), and the teeth were embedded in the center of the ring so that the labial surface remained exposed and facing upward. The buccal surfaces were polished by Sof-Lex Disks (3M ESPE, LA, CA, USA) progressively (beginning with the coarse, then medium and fine, ending with the superfine) using a contra-angle slow-speed handpiece (NSK, Saitama, Japan) (16). Then, on the buccal surface of each tooth, a circular window of 6mm in diameter was standardized. After that, a circle of adhesive tape of 6 mm in diameter was cut out and positioned on the buccal surface of the tooth. All of the samples received two layers of nail varnish (China). Then, the adhesive tape was removed, leaving a window on the tooth’s buccal surface.

-Randomization

An independent person adopted simple randomization was utilized in this study to divide the study samples randomly into three major groups based on the type of treatment: untreated control group, APF gel group, and Er, Cr: YSGG+ 1.23%APF gel group using the “Random sequence Generator” tool (https://www.random.org/ sequences).

-Experimental design of the study

1. Group I: Untreated group where the samples were kept in deionized water that was replaced daily without any treatment.

2. Group II: APF1.23 % (pH: 3.6–3.9, Keystone Industries, Gibbstown, NJ, USA) was applied for 4 minutes with a cotton bud according to the manufacturer’s instructions, and then the gel was removed with cotton rolls and washed with deionized water.

3. Group III: The specimens were exposed to a 2,780 nm pulsed Er, Cr: YSGG laser (Waterlase iPlus Biolase, San Clemente, CA, USA) with the following settings: 0.75 W of power, 60 s pulse duration, 20-hertz repetition frequency, air pressure was 11%, and the water level was 0%. The samples were irradiated in a scanning style for ten seconds, followed by fluoride gel application for four minutes. MZ6 tip was used in this study. The tip was parallel to the enamel’s surface and 1 mm away from it. This was performed using an elastic band to attach an endodontic file with a rubber stopper to the handpiece to maintain a distance of 1 mm from the surface during each treatment to create a constant spot size. All specimens were kept in deionized water at room temperature.

 The two examiners who performed data measurements and the statistician were blinded to the surface treatment method. The groups were only revealed at the end of the measuring and data analysis process.

-Demineralization 

To induce initial erosion, the samples were placed separately in 20 ml of carbonated soft drink (Pepsi, Baghdad, Iraq) for 2 minutes. Then they were washed with deionized water and kept within it for six hours until the next cycle. Four consecutive cycles of immersion were performed at six hours intervals. Carbonated soft drink and deionized water were refreshed for each cycle.

-Microhardness evaluation 

The microhardness of the enamel surface was measured at baseline, following treatment, and after demineralization. The microhardness indicates the mechanical properties of the teeth, as measured by a digital microhardness tester (Laryee Technology Ltd, Beijing, China) at 500 gm load for 30 seconds. For each specimen, three indentations were made, and three records were obtained. The mean was then calculated for these three recordings. The Microhardness test was done using an optical microscope, using a square-based diamond indenter with an angle of 136˚ between the opposed faces. The magnification used was X50.

The same examiner using the same calibrated machine, took all the measurements. The average of the three indentations in each reading was taken, and that represents the microhardness value for each specimen (17).

-SEM analysis 

Representative one sound specimen and one specimen from each study group (control, APF gel only, and combined Er, Cr: YSGG laser +1.23% APF) were prepared for scanning electron microscopy (SEM) analysis (Thermo Fisher Scientific, Waltham, USA). The samples were dried, then sputter coated with gold by placing them in a vacuum system coating machine (Ngstrom Advanced, ion sputter), where they were steamed with a layer of gold about (10-15) nm to establish a conductive metal layer on the sample, to restrict charging, minimize heat damage, and maximize the secondary electron signal necessary for SEM topographic analysis. After gold coating, representative samples were ready for SEM analysis at magnifications ×100, ×1000, and ×2000 (18).

-Statistical Analysis 

Data description, analysis, and presentation were performed using Statistical Package for Social Science (SPSS version -22, Chicago, Illinois, USA). Shapiro–Wilk test was used for the assessment of data normality. Data analyses were performed by Levene test and General Linear Model Repeated Measure Factorial ANOVA with Bonferonni post hoc test; the accepted level of significance was 0.05%.

## Results

-Microhardness

Shapiro Wilk test showed that surface microhardness is normally distributed among phases and groups at *p*>0.05.

Results of the present study showed a non-significant difference in the values of microhardness between groups at baseline (*p*=0.472). After treatment, the untreated control group had the lowest microhardness value while group III had the highest mean of the microhardness value with a statistical significant difference (*p*=0.000), but there was no statistical significance from group II (*p*=0.643).

After demineralization with Pepsi cola drink, the control group had been demineralized to a greater extent. It showed the lowest value of microhardness with a mean of 184.274, followed by the group using APF gel with a mean of 262.652. However, the group of combined laser and APF gel had the highest score of the microhardness value, with a statistical significant difference between groups, as shown in Table 1.

**Table 1:**
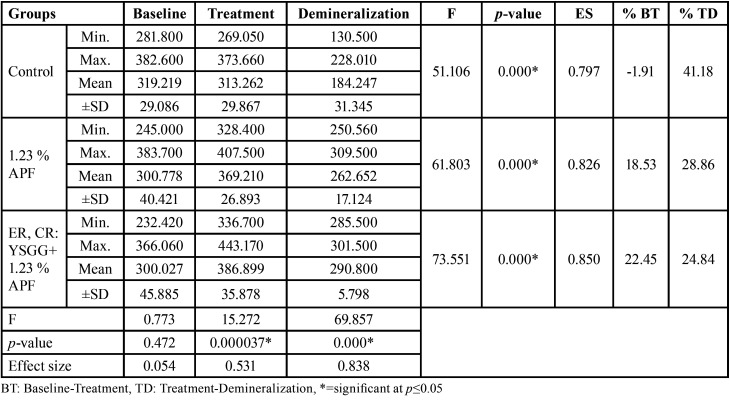
Comparison of microhardness values using ANOVA test among phases and groups.

Table 2 shows the multiple pairwise comparisons of surface microhardness among phases of the groups using the Bonferroni post hoc test. The results showed that there was a statistical significance difference among phases (baseline, treatment, demineralization), except for the control group (between the baseline and treatment) and group III (between the baseline and demineralization), no statistical significant difference was found. Meanwhile, Multiple pairwise comparisons showed that there were statistical significant differences among groups in the treatment phase and the demineralization phase, except between the fluoride gel group and combined laser fluoride group (*p*=0.643) in the treatment phase, as shown in Table 3.

**Table 2:**
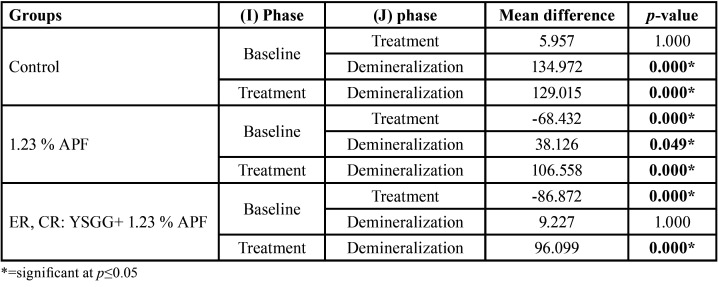
Multiple Pairwise Comparisons of Surface microhardness among phases by groups using Bonferroni post hoc.

**Table 3:**
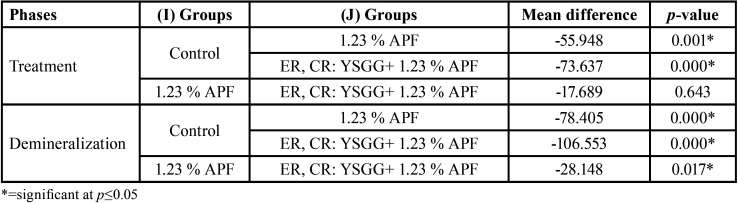
Multiple Pairwise Comparisons of surface microhardness among groups in treatment and demineralization phase using Bonferroni post hoc.

-Scanning electron microscopy

**Figure 1:**
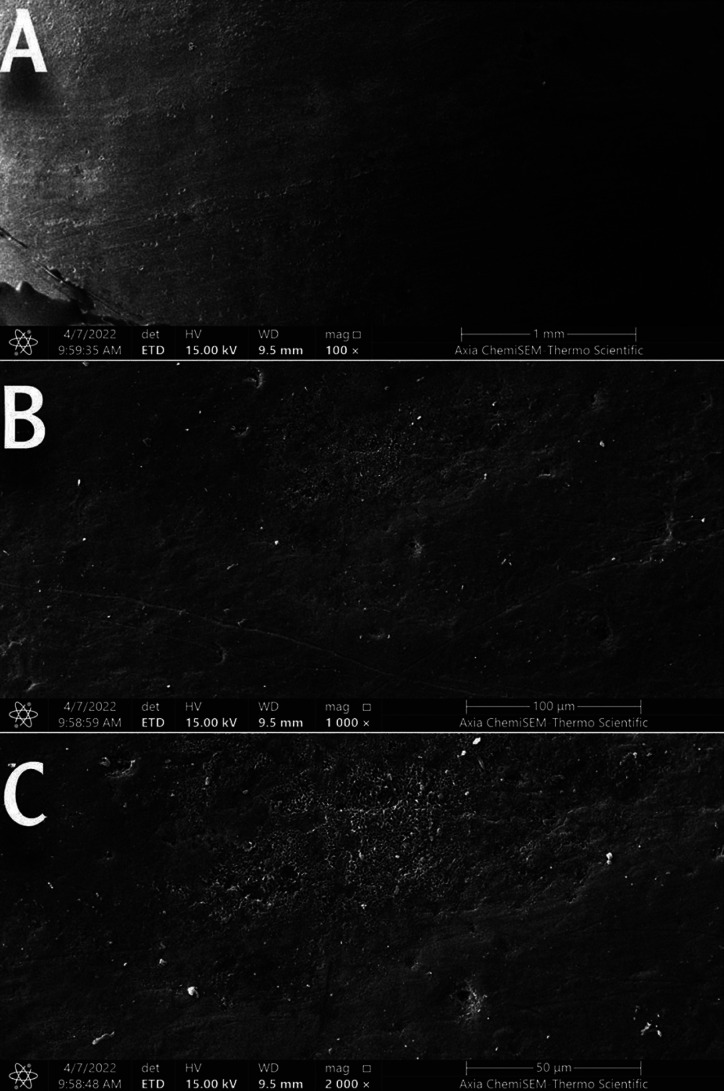
Scanning electron micrograph of the sound enamel surface. Magnification 100 (A), 1000 (B), and 2000 (C).

**Figure 2:**
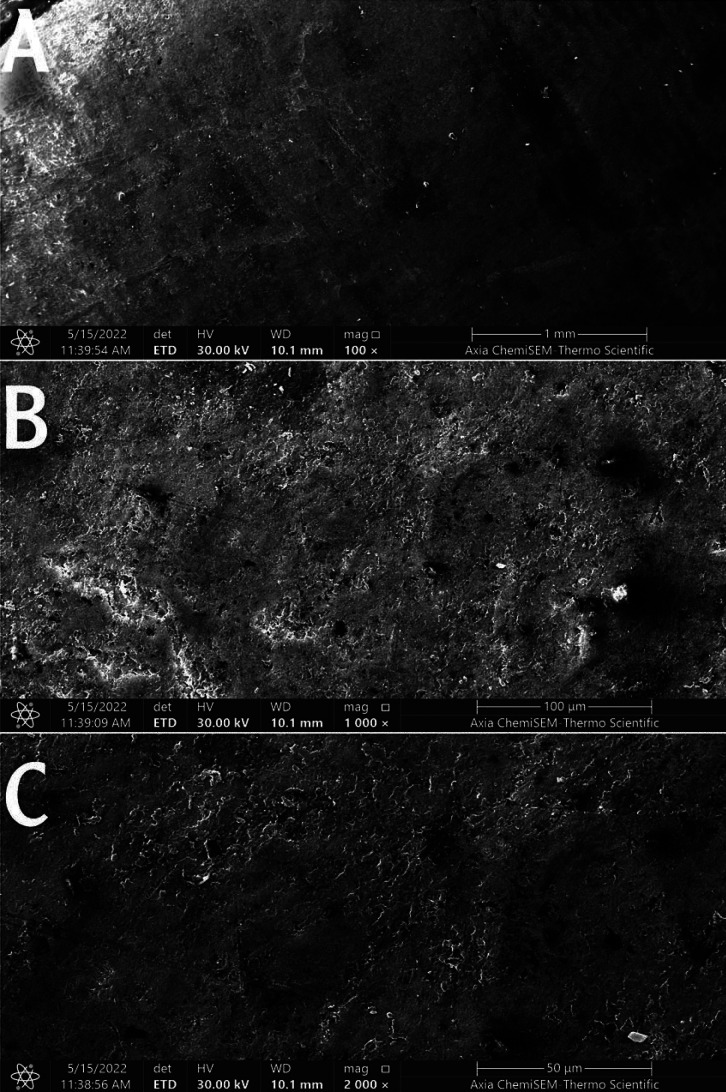
Scanning electron micrograph of the control enamel surface. Magnification 100 (A), 1000 (B), and 2000 (C).

**Figure 3:**
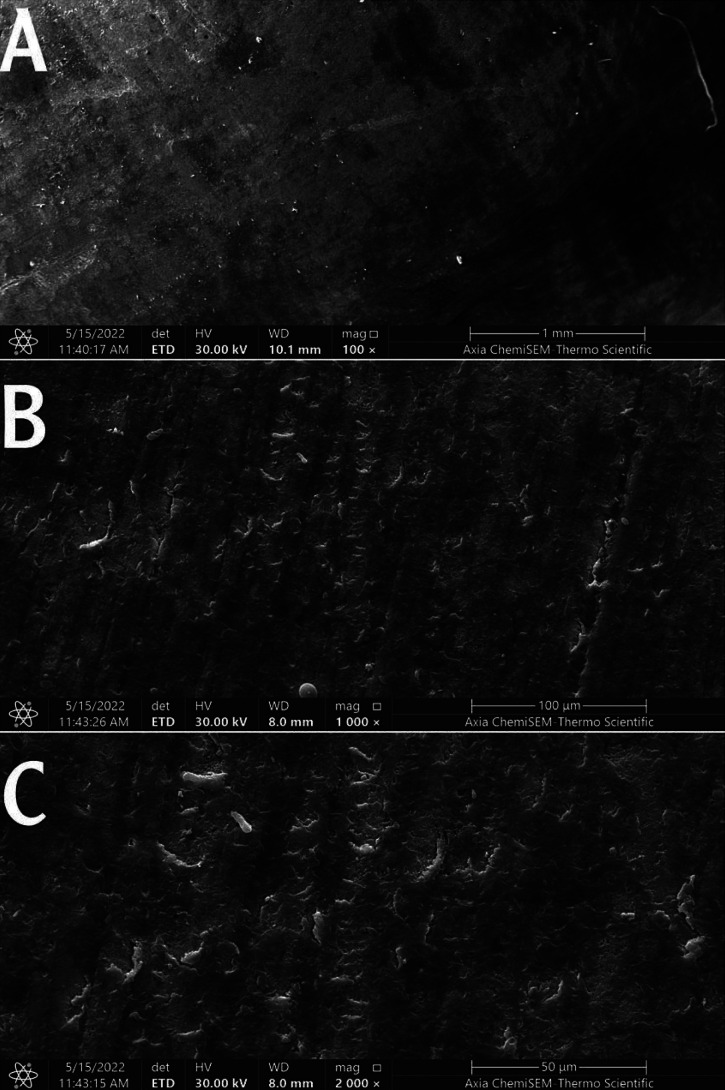
Scanning electron micrograph of the enamel surface treated with 1.23% APF. Magnification 100 (A), 1000 (B), and 2000 (C).

**Figure 4:**
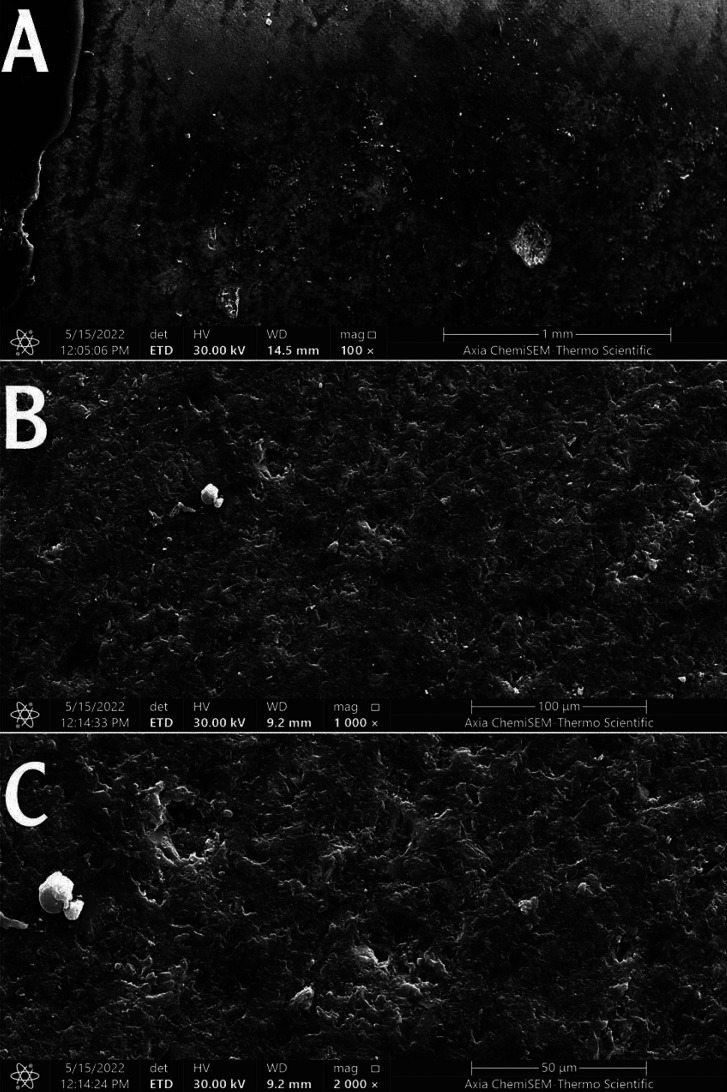
Scanning electron micrograph of the enamel surface treated with Er, Cr: YSGG laser and 1.23% APF. Magnification 100 (A), 1000 (B), and 2000 (C).

1. Sound enamel sample: SEM examination of the polished sound untreated enamel surface showed a flattened, smooth surface with multiple enamel prism ends and the absence of cracks and surface deposits, Figure 1.

2. Control group sample: The SEM image of the untreated control enamel surface after demineralization revealed a microscopic alteration of the enamel surface presented as coalescing of enamel rods with a destructive aspect, including irregularities and porosities throughout the enamel surface, exposed prisms due to enamel acid dilution. The acid demineralization resolved the untreated surface, and enamel prisms consistently presented with a honeycomb appearance after demineralization, Figure 2.

3. 1.23% APF sample showed partially dissolved enamel, globular deposits of fluoride on the enamel surface and CaF2 deposits, as shown in Figure 3.

4. Er, Cr: YSGG and APF sample: SEM image showed that the surface defects produced by laser radiation were reconstructed by fluoride deposition; CaF2 globules embedded between crystals, melting and fusion of crystals, areas of microporosities, and partially dissolved enamel surfaces were also observed, Figure 4.

## Discussion

Enhancing enamel resistance with laser is considered as a revolution technology for preventing dental caries and erosion. Enamel crystals will be melted and fused due to the laser’s photothermal effect; making the enamel more acid-resistant. Researchers had also stated that changes in surface resistance could also be due to the laser photochemical effect through a decrease in the carbonate concentration or partial decomposition of the organic matrix. However, it is essential to study the effectiveness of Er, Cr: YSGG in combination with 1.23% APF in protecting enamel from erosion.

In addition, a comparison of these treatments’ protective effects had not been established, because there are still several conflicting views on the effects on the structure of tooth enamel, this is most likely because multiple factors are involved such as power, pulse frequency, and irradiation duration (19).

When Er, Cr: YSGG is employed at a sub-ablative wavelength, the temperature will rise without heating the nearby tissues, which may cause enamel to modify crystallographically and become more acid-resistant (20). To protect the enamel from mechanical damage, the irradiation parameters used in this study were intended to be below the ablation threshold. So, 0.75 W was used in this study based on the prior experiments that tested this power with various Er, Cr: YSGG power levels or with other types of lasers. The results showed that 0.75 W was the best power for increasing acid resistance (21-24).

Although water functions as a cooler when operating lasers for protecting the tooth and the adjacent tissues from thermal, the presence of water when the laser interacts with the dental hard tissues is an essential factor that promotes ablation (25). But many other studies used the air/water cooling parameters to keep the temperature from rising too much above the ablation threshold or damaging the pulpal tissue (26,27).

In the present research, the enamel surface was first exposed to laser irradiation before fluoride gel was applied. This was in accordance with many recent studies, which stated that it is better to use a laser first than a gel as it may act as a barrier coat to laser action (28).

In this study, the effects of Er, Cr: YSGG laser and APF gel on permanent human enamel resistance to demineralization was studied *in vitro* using the Vickers microhardness test and scanning electron microscopy (SEM). The microhardness test can investigate the effect of extrinsic and intrinsic acids on dental hard tissues. This technique can assess the early phases of enamel and dentin disintegration, which are accompanied by surface weakening and softening (18).

Results of this study revealed that the microhardness value at the baseline between groups did not show any statistical significance difference. However, after treatment, Fluoride treatment significantly enhanced the microhardness of the enamel, and there was a statistical increase in the microhardness in group II (fluoride treatment only) and group III (laser and fluoride), with the highest microhardness score value in group III. This was consistent with the results of other studies that used fluoride alone (27). Meanwhile, the results (regarding group III) are also consistent with the results reported by de Freitas *et al*., and Moslemi *et al*., who claimed that fluoride treatment plus this type of laser treatment reduced enamel demineralization more effectively than fluoride treatment alone (20,23).

Fekrazad and Ebrahimpour reported a significant difference between the calcium content of the groups treated with fluoride and a combination of acidulated phosphate fluoride gel (APF) and Er, Cr: YSGG with the control group. They also found that a combination of Er, Cr: YSGG laser and fluoride or fluoride alone resulted in significantly lower enamel solubility than the application of laser alone (29).

In contrast, the results of the present study were inconsistent with Ulusoy *et al*., who found that the coapplication of fluoride and laser had no additive effects. This might be because of the application of fluoride first, then laser, which may act as a barrier coat (30).

A scanning electron microscope was used to observe the structural analysis. It is commonly used to study the effects of different surface treatments on enamel morphology (18,31). Consequently, a scanning electron microscope was utilized in this study. SEM analysis in the present study showed a smooth homogenous surface with a uniform rodless enamel surface layer of the sound specimen. This was consistent with Huang *et al*., and El Moshy *et al*., (32,33).

A dissolving and porous enamel surface was observed by SEM concerning the sample with Group I (untreated enamel) after pH cycling with a soft drink. Belcheva *et al*., who revealed numerous pores on the surface of the etched enamel, reported the same findings (18).

In Group II ( treated with 1.23 % APF), morphological changes were observed by partially dissolved enamel, and the presence of amorphous, globular, and crystalline structures and CaF2 deposits were also found, but this accumulation did not show a homogeneous distribution. These findings were in accordance with that of other studies (15).

In Group III (treated with laser and 1.23% APF gel), CaF2 globules embedded between crystals were observed. The crystals were densely packed and emerged through pores produced by laser action. Irradiated enamel surfaces had undergone morphological changes that increased their resistance to demineralization. The surface granular and globular material coatings might provide a reservoir of mineral phases during the acid attack. The surface layer contains a higher quantity of fluoride in the form of calcium fluoride achieved by the reaction of fluoride gel with the enamel surface, making it more resistant to erosion. These were the same findings observed by Kumar *et al*., (34).

Laser irradiation induces chemical and morphological surface modifications. Chemical changes originate from the elimination of carbonated apatite, while changes in morphology were caused by an increase in surface temperature. These changes increase fluoride uptake at the tooth’s surface after applying fluoride gel, increasing the protection of the enamel (35).

Ana *et al*., noted an increase in the formation of calcium fluoride-like material on the enamel surface after exposing the tooth to Er, Cr: YSGG laser irradiation before a fluoride application, which could explain why this combination treatment was able to control the enamel erosion in the present study (36).

The present experimental findings indicated some positive possibilities with some clinical limitations. Additional studies are needed to determine the amount of mineral loss, the laser parameters that produce the best results over various techniques, and the efficient sequence for applying laser and fluoride together (i.e., whether to use the laser first, last, or throughout fluoride application).

## Conclusions

Within the limitations of this study, the application of fluoride alone as well as the combination of Er:Cr: YSGG laser (0.75 W) followed by fluoride application had improved the microhardness of the enamel surface and enhanced the protection of tooth enamel against erosion, but the treatment with combined laser and fluoride had the superiority over the fluoride application alone.
